# Association among resilience, post-traumatic stress disorder, and somatization in frontline healthcare workers in COVID-19: The mediating role of perceived stress

**DOI:** 10.3389/fpsyt.2022.909071

**Published:** 2022-09-16

**Authors:** Minjie Li, Xingfeng Yu, Dan Wang, Ying Wang, Lipei Yao, Yunmiao Ma, Xiaomei Liu, Yulian Zhang

**Affiliations:** ^1^Department of Nursing, Shaanxi Provincial People’s Hospital, Xi’an, China; ^2^Director’s Office, Shaanxi Provincial People’s Hospital, Xi’an, China

**Keywords:** China, COVID-19, frontline healthcare workers, mediation, perceived stress, PTSD, resilience, somatization

## Abstract

**Background:**

Frontline healthcare workers were at a high risk of infection and developing mental health problems during the outbreak of coronavirus disease 2019 (COVID-19). It is important to monitor the symptoms of post-traumatic stress disorder (PTSD) and somatization among frontline healthcare workers in China.

**Aim:**

This study aimed to investigate PTSD, somatization, resilience, and perceived stress among frontline healthcare workers fighting against COVID-19 and examine the mediating effects of perceived stress on resilience in both PTSD and somatization.

**Methods:**

The study was conducted from December 2021 to February 2022 through an online survey of frontline healthcare workers fighting against COVID-19. The survey included questions regarding socio-demographic information, resilience (10-item Conner–Davidson Resilience Scale, CD-RISC-10), perceived stress (14-item Perceived Stress Scale, PSS), PTSD (Checklist-Civilian Version, PCL-C), and somatization (Symptom Checklist-90). The PROCESS macro for SPSS was used to examine the mediating effects of perceived stress.

**Results:**

Approximately 14.9% of healthcare workers had possible PTSD (PCL-C score of ≥ 44), and 41.04% of the workers had low resilience (CD-RISC-10 score of ≤ 25.5). Approximately 54.05% of healthcare workers were symptomatic, and 14.7% had a moderate or higher degree of somatization with sleep-related problems as the most common symptom. Perceived stress was negatively correlated with resilience (*r* = –0.527, *p* < 0.001) and positively correlated with PTSD (*r* = 0.505, *p* < 0.001) and somatization (*r* = 0.361, *p* < 0.001). In addition, perceived stress mediated the relationship between resilience and PTSD [indirect *b* = –0.382; bootstrapped confidence interval (CI), –0.454, –0.319] and somatization (indirect effect *b* = –0.159; bootstrapped CI, –0.199, –0.123).

**Conclusion:**

The prevalence of PTSD and somatic symptoms indicates that the mental health of frontline healthcare workers deserves more attention. Resilience is negatively associated with PTSD and somatization, and the relationship among resilience, PTSD, and somatization is mediated by perceived stress. Strategies for reducing perceived stress and increasing resilience may help to prevent and alleviate PTSD and somatization.

## Introduction

Frontline healthcare workers were at a huge risk of infection during the coronavirus disease 2019 (COVID-19) pandemic (1). According to the World Health Organization (WHO), healthcare workers accounted for 14% of all infected cases worldwide. According to the WHO–China Joint Mission, approximately 3387 healthcare workers were reported to have COVID-19 in China as of February 20, 2020 (2). The pandemic imposed an enormous psychological burden on Chinese frontline medical staff involved in the treatment of patients with COVID-19.

The COVID-19 pandemic posed the risk of post-traumatic stress disorder (PTSD) and somatization to frontline healthcare workers who are constantly involved in treating patients and risk populations (3). PTSD is a mental health condition triggered by a terrifying past event–either experiencing or witnessing it (4). An umbrella review reported that the prevalence of PTSD was high (13.52%) among frontline healthcare workers during the COVID-19 pandemic (5). Resilience acts as a buffer against PTSD (6, 7) and is usually defined as the successful adaptation to adversity, trauma, tragedy, threats, or significant sources of stress (8). Building resilience can help healthcare workers to better cope with and recover from adverse events to achieve better wellbeing (9–11). The scale used for measuring resilience in this study involves certain aspects of coping, such as adapting to changes, dealing with any circumstance, and considering the humorous side of problems (12). Successful management of stressful situations usually requires active coping, ways to diminish negative emotions, a positive outlook, moral compass, good social support, and cognitive flexibility, which are psychosocial factors of resilience that protect against and aid recovery from post-traumatic stress (13). Perceived stress, the feelings or thoughts that individuals have about how much stress they are experiencing during a specific period (14), is one of the psychological susceptibility factors of PTSD (15). Resilience appears to be a determinant of perceived stress; decreased resilience is related to a higher perceived stress level and the increased number and intensity of stressful life events (16). A study on Korean firefighters reported that those with higher levels of resilience overcame adversity and were protected from the risk of developing the symptoms of PTSD, whereas those with lower levels of resilience were more susceptible to the development of PTSD, and these changes were mediated by perceived stress (17). Therefore, we propose the following hypothesis:

*Hypothesis 1: During the COVID-19 pandemic, resilience buffered the effects of life events on PTSD through the mediating effects of perceived stress among frontline healthcare workers.*

Somatization is a form of mental illness that causes multisystem physical symptoms, which may or may not be traceable to a physical cause (18). Massive negative information and negative emotions often produce various somatic symptoms. A study reported that the prevalence rate of somatization among healthcare workers was 9.59%, which was higher than that among non-healthcare workers (19). PTSD is associated with somatic problems, such as unexplained dizziness, tinnitus, and blurry vision, and several medical conditions, including cardiovascular, respiratory and gastrointestinal disorders (20). Somatic symptoms severely deteriorate the mental health of healthcare workers and compromise the competence to fight against COVID-19.

Evidence suggests that an inverse relationship was observed between somatization and resilience in the general population during the COVID-19 pandemic; however, higher somatization was associated with increasing levels of perceived stress (21). Perceived stress is the cognitive appraisal of the degree of pressure and the ability to cope with objective stressors (22). Individuals with high resilience are more resourceful in coping with stress and less overwhelmed by stressors and usually have lower levels of perceived stress, which may eventually alleviate somatization (16, 21). Therefore, we speculate that perceived stress plays a role in the relationship between resilience and somatization and hence propose the following hypothesis:

*Hypothesis 2: During the COVID-19 pandemic, resilience buffered the effects of life events on somatization through the mediating effects of perceived stress among frontline healthcare workers.*

Exploring the mediating role of perceived stress in the association between resilience and PTSD/somatization is important for preventing and alleviating PTSD and somatization among frontline healthcare professionals. If perceived stress is established as a mediator, it can be useful for making regulatory policies and developing intervention programs considering the role of perceived stress and resilience in reducing PTSD and somatization among frontline healthcare professionals. Perceived stress is reported to have mediating effects on resilience and somatization in firefighters (17). However, to the best of our knowledge, the mediating role of perceived stress in resilience and PTSD/somatization has not been reported in frontline healthcare professionals involved in the treatment of patients with COVID-19. Therefore, this study aimed to examine the mediating role of perceived stress in resilience and PTSD/somatization based on the two abovementioned hypotheses.

## Materials and methods

### Design

An online cross-sectional survey design was adopted to query frontline healthcare workers involved in the treatment of patients with COVID-19.

### Sample

Frontline healthcare professionals involved in the management of COVID-19 from December 2021 to January 2022 were recruited. The inclusion criteria were as follows: frontline healthcare workers who were involved in treating patients with COVID-19, were in contact with diagnosed patients with COVID-19 and undiagnosed individuals in whom COVID-19 cannot be ruled out, working in designated COVID-19 hospital isolation wards and undesignated COVID-19 hospital closed-loop management wards, and involved in community nucleic acid testing for COVID-19.

### Measures

Data regarding sociodemographic characteristics (age, sex, and educational qualification) and symptoms (such as trouble falling asleep and fatigue) were recorded, and the following questionnaires related to PTSD, somatization, resilience, and perceived stress were presented to all participants in an online survey.

#### Post-traumatic stress disorder checklist-civilian version

The Post-traumatic Stress Disorder Checklist-Civilian Version (PCL-C) is a standardized self-report rating scale for PTSD symptoms closely based on the DSM-IV criteria. The PCL-C comprises 17 items that correspond to the key symptoms of PTSD and is usually applied to any traumatic event and population. The scale consists of the following four dimensions: intrusive re-experience of symptoms, avoidance/numbing of symptoms, social function impairment, and alarmed responses/hyperarousal. The questions were worded generically to refer to “stressful experiences in the past” and could be scored to provide a presumptive diagnosis of PTSD, screen individuals for PTSD, and monitor changes in symptoms during and after treatment. This version of the scale simplifies assessment based on multiple traumas because symptom endorsements are not attributed to a specific event. Therefore, this tool can help to assess whether an individual may require further assessment or treatment for PTSD. In this study, respondents indicated how much they have been bothered by a symptom over the past month using a 5-point scale, ranging from 1 (not at all) to 5 (extremely). Scores of ≥ 44 indicated possible PTSD (23), and Cronbach’s alpha value of the scale was 0.966.

#### Somatization subscale of symptom Checklist-90

The Symptom Checklist-90 (SCL-90) scale currently used in China was translated by Wang (24). In this study, the scale was used to assess the complaints of discomfort in cardiovascular, gastrointestinal, respiratory, and other systems and other somatic behaviors including headache, backache, muscle ache, and anxiety. A 5-point scale was used, with 1 indicating no symptoms and 5 indicating severe symptoms. The mean value plus one standard deviation was used as the measurement standard to define participants with a moderate or higher degree of somatization (25), and the cut-off value was 25.71.

#### Conner–Davidson resilience scale

The 10-item Conner–Davidson Resilience Scale (CD-RISC-10) was used to measure resilience, with a higher score indicating better resilience. The scale has been validated in a Chinese population who survived an earthquake, with good internal consistency (Cronbach’s alpha = 0.91) and test–retest reliability (*r* = 0.90 for a 2-week interval) (26), and in Chinese undergraduates and patients with depression with favorable criterion-related validity (27). A cut-off value of 25.5, which was previously reported in Chinese parents whose children were undergoing cancer treatment, was used in this study (28).

#### Perceived stress scale

Perceived stress was measured using the 14-item Perceived Stress Scale (PSS), which assesses the degree to which participants have perceived a situation in their life within a specific period as stressful. Participants were asked to report how they felt and thought during the previous 7 days. Each item was scored ranging from 0 (never) to 4 (always), and the overall score ranged from 0 to 56, with a higher score indicating higher perceived stress levels. The Chinese version of PSS-14 has demonstrated favorable psychometric properties (Cronbach’s alpha = 0.78) as a self-administered inventory for evaluating stress levels in the Chinese population (29).

### Procedure

Data were collected from December 2021 to February 2022. A poster with a QR code was forwarded by the leadership of all departments, which could be scanned to access the online questionnaire. Data were collected based on an anonymous self-administered questionnaire using a secure survey website, “Wenjuanxing”.^1^

### Statistical analysis

The SPSS Statistics (version 23.0) software (IBM Corp., Armonk, New York) was used for statistical analysis. Continuous variables with normal distribution were expressed as the mean ± SD, whereas those with non-normal distribution were expressed as the median and interquartile range (IQR). Normality was assessed *via* a statistical test with the skewness and kurtosis value in the range of –/ + 2. Categorical variables were expressed as frequencies (proportion) to examine the characteristics and prevalence of stress, resilience, PTSD, and somatization. Univariate analysis was performed to examine the relationship between PTSD and somatization. Continuous variables were assessed *via* Pearson (or Spearman if required) correlation analysis to examine the relationship among different variables. Scatter plots of study variables were constructed, and a *p*-value of < 0.05 (two-tailed) was considered significant.

Bootstrapping is a statistical method that used random resampling with replacement to estimate a population parameter. It is the state-of-the-art method for testing indirect effects in mediation models in which the data distribution is usually asymmetric (30). It does not require the assumption of normality of the distribution (31). The PROCESS macro v4.0 for SPSS was used to test the mediating effects (32). To determine the presence of mediating effect, the indirect effects of a predictor (resilience) on outcomes (PTSD and somatization) through a mediator (perceived stress) should be significant. Model 4 was used in PROCESS for mediation analysis, and 5,000 resamples were made for estimating the bootstrapped 95% confidence interval (CI). The mediation effect was considered significant if the 95% CI does not include 0 (zero).

### Ethical considerations

Ethical approval was obtained from the local hospital Ethics Committee (reference number: 2022R019). Informed consent was obtained digitally after introducing the online survey. The anonymity and confidentiality of all information were guaranteed during data collection.

## Results

A total of 953 healthcare workers that met the inclusion criteria were included in the study. After omitting straight-lining responses, 938 participants were included in the final analysis.

### Descriptive statistics

The participant cohort comprised 873 nurses, 37 doctors, 22 medical technicians, and 6 supporting staff members. The mean age of participants was 31.10 years (*SD* = 5.77). Most participants were women (93.71%) and had primary and middle titles (97.55%). Most participants worked with 5.65 (*SD* = 1.73) shifts per week. The mean professional experience working in a hospital was 8.90 years (*SD* = 5.97) (Table 1).

**TABLE 1:** Sociodemographic information of participants (*n* = 938) and study variables.

Variable	M (*SD*)/*n* (%)	Variable	M (*SD*)/*n* (%)
Age (years)	31.10 ± 5.77	Work schedules/week	Median (IQR) 6 (5–7)
Sex		Working site	
Men	59 (6.29)	Designated hospital wards for patients with COVID-19	311 (33.16)
Women	879 (93.71)	Undesignated hospital closed-loop management wards for patients at risk for COVID-19	89 (9.49)
Marital status		Community nucleic acid testing	538 (57.36)
Unmarried	318 (33.9)	Work content	
Married	606 (64.61)	Clinical treatment/caring	407 (43.39)
Other	14 (1.49)	Other work on the frontline	531 (56.61)
Educational qualification		Provision of support to Hubei in 2019	
College diploma or lower	273 (29.1)	Yes	71 (7.57)
University diploma	654 (69.72)	No	867 (92.43)
Master’s degree or higher	11 (1.17)	Training on the treatment and caring of patients with COVID-19	
Monthly income per capita (RMB)	Median (IQR) 4,000 (3,000–5,000)	Yes	890 (94.88)
Parity		No	48 (5.12)
Yes	562 (59.91)	Competence in position	
None	376 (40.09)	Good	845 (90.09)
Technical title		Fair	91 (9.7)
Primary	625 (66.63)	Poor	2 (0.21)
Middle	290 (30.92)	Mental support	
Vice-senior	21 (2.24)	Yes	667 (71.11)
Senior	2 (0.21)	No	271 (28.89)
Staff		Frequency of contact with family and friends	
Physician	37 (3.94)	Always	175 (18.66)
Nurse	873 (93.07)	Usually	287 (30.6)
Medical technician	22 (2.35)	Often	310 (33.05)
Supporting staff	6 (0.64)	Occasionally	110 (11.73)
Ways of COVID-19-related work		Rarely	56 (5.97)
Volunteer	726 (77.4)	Psychological support from family and friends	
Designated	212 (22.6)	Yes	880 (93.82)
Years of work	8.90 ± 5.97	No	58 (6.18)

IQR, interquartile range (Q1–Q3: 25–75%).

### Study variables and univariate analysis

As shown in Table 2, among the 938 participants, 140 (14.9%) had possible PTSD, and 385 (41.04%) had low resilience. Approximately 14.7% of participants had a moderate or higher degree of somatization, and 54.05% of participants were symptomatic. The five most common symptoms were trouble falling asleep (32.56%), fatigue, insomnia, inability to wake up early, and muscle soreness. The results of univariate analysis revealed that healthcare professionals who volunteered to work on the frontline, were engaged in non-clinical treatment/management, self-assessed to be competent in their position, had frequent contact with family and friends, and received psychological support from family and friends were less likely to develop PTSD (*p* < 0.05). However, healthcare professionals who did not volunteer to work on the frontline, did not receive training on the treatment and care of patients with COVID-19, were not competent in their position, did not have mental support, less frequently interacted with family and friends, and did not receive psychological support from family and friends were more like to have a moderate or higher degree of somatization (*p* < 0.05) (Table 3).

**TABLE 2:** Variables and symptoms among frontline healthcare professionals (*n* = 938).

Variables/Symptoms	*n* (%)	Variables/Symptoms	*n* (%)	Variables/Symptoms	*n* (%)
PTSD	140 (14.93)	Constipation	136 (14.50)	Diarrhea	44 (4.69)
Moderate or higher degree of somatization	138 (14.71)	Loss of appetite	122 (13.01)	Sweat	43 (4.58)
Low resilience	385 (41.04)	Menstrual disorder^#female^	120 (13.65)	Muscle tension	41 (4.37)
Symptomatic	507 (54.05)	Irritability	109 (11.62)	Nausea	41 (4.37)
Trouble falling asleep	296 (31.56)	Indigestion	91 (9.70)	Heart palpitations	39 (4.16)
Fatigue	288 (30.70)	Stomachache	83 (8.85)	Muscle weakness	32 (3.41)
Insomnia	284 (30.28)	Endocrine disorders	76 (8.10)	Changes in dietary preferences	25 (2.67)
Inability to wake up early	253 (26.97)	Skin allergy	68 (7.25)	Shortness of breath	20 (2.13)
Muscle soreness	178 (18.98)	Body ache	54 (5.76)	Overreaction	18 (1.92)
Headache	163 (17.38)	Rhinitis pharyngitis	49 (5.22)	Fever	10 (1.07)
Anxiety	156 (16.63)	Tinnitus	47 (5.01)	Abnormal behavior	2 (0.21)
Dizziness	151 (16.10)	Somatosensory abnormality	47 (5.01)	Smell-related disorders	1 (0.11)

^#^Only for female.

**TABLE 3:** Univariate analysis for PTSD and a moderate or higher degree of somatization (*n* = 938).

Variable	*n*/% (PTSD/somatization)	χ^2^ (PTSD/somatization)	Variable	*n*/% (PTSD/somatization)	χ^2^ (PTSD/somatization)
**Age (years)**			**Work schedules/week**		
≤30 (*n* = 490)	63 (12.9)/73 (14.9)	3.460/3.504	<6 (*n* = 400)	64 (16.0)/51 (12.8)	0.634/2.140
31–40 (*n* = 385)	66 (17.1)/51 (13.2)		≥6 (*n* = 538)	76 (14.1)/87 (16.2)	
>41 (*n* = 63)	11 (17.5)/14 (22.2)		**Working site**		
**Sex**			Designated hospital wards for patients with COVID-19 (*n* = 311)	42 (13.5)/33 (10.6)	5.922/8.947*
Men (*n* = 59)	9 (15.3)/9 (15.3)	0.005/0.015	Undesignated hospital closed-loop management wards for patients at risk for COVID-19 (*n* = 89)	21 (23.6)/20 (22.5)	
Women (*n* = 879)	131 (14.9)/129 (14.7)		Community nucleic acid testing (*n* = 538)	77 (14.3)/85 (15.8)	
**Marital status**			**Work content**		
Unmarried (*n* = 318)	39 (12.3)/42 (13.2)	0.092/0.476 (Both Fisher *p*)	Clinical treatment/caring (*n* = 407)	73 (17.9)/60 (14.7)	5.132*/0.001
Married (*n* = 606)	97 (16.0)/93 (15.3)		Other work on the frontline (*n* = 531)	67 (12.6)/78 (14.7)	
Other (*n* = 14)	4 (28.6)/3 (21.4)		**Provision of support to Hubei in 2019**		
**Educational qualification**			Yes (*n* = 71)	5 (7.0)/13 (18.3)	3.759/0.792
College diploma or lower (*n* = 273)	34 (12.5)/43 (15.8)	5.526/0.466	No (*n* = 867)	135 (15.6)/125 (14.4)	
University diploma (*n* = 654)	102 (15.6)/93 (14.2)		**Training on the treatment and caring of patients with COVID-19**		
Master’s degree or higher (*n* = 11)	4 (36.4)/2 (18.2)		Yes (*n* = 890)	129 (14.5)/126 (14.2)	2.544/4.267*
**Monthly income per capita (RMB)**			No (*n* = 48)	11 (22.9)/12 (25.0)	
≤3,000	61 (14.7)/72 (17.3)	0.044/5.783	**Competence in position**		
3,001–5,000	47 (15.3)/44 (14.3)		Good (*n* = 845)	113 (13.4)/109 (12.9)	17.536**/23.905**
>5,000	32 (14.9)/22 (10.2)		Fair (*n* = 91)	27 (29.7)/29 (31.9)	
**Parity**			Poor (*n* = 2)	0	
Yes (*n* = 562)	91 (16.2)/82 (14.6)	1.772/0.016	**Mental support**		
None (*n* = 376)	49 (13.0)/56 (14.9)		Yes (*n* = 667)	93 (13.9)/87 (13.0)	1.755/5.123*
**Technical title**			No (*n* = 271)	47 (17.3)/51 (18.8)	
Primary (*n* = 625)	80 (12.8)/87 (13.9)	7.690/0.697 (Fisher *p*)	**Frequency of contact with family and friends**		
Middle (*n* = 290)	57 (19.7)/48 (16.6)		Always (*n* = 175)	15 (8.6)/19 (10.9)	19.495**/16.148**
Vice-senior (*n* = 21)	3 (14.3)/3 (14.3)		Usually (*n* = 287)	32 (11.1)/28 (9.8)	
Senior (*n* = 2)	0		Often (*n* = 310)	59 (19.0)/58 (18.7)	
**Staff**			Occasionally (*n* = 110)	26 (23.6)/24 (21.8)	
Physician (*n* = 37)	4 (10.8)/6 (16.2)	0.486/0.856 (Both Fisher *p*)	Rarely (*n* = 56)	8 (14.3)/9 (16.1)	
Nurse (*n* = 873)	131 (15.0)/129 (14.8)		**Psychological support from family and friends**		
Medical technician (*n* = 22)	3 (13.6)/2 (9.1)		Yes (*n* = 880)	121 (13.8)/117 (13.3)	15.484**/22.764**
Supporting staff (*n* = 6)	2 (33.3)/1 (16.7)		No (*n* = 58)	19 (32.8)/21 (36.2)	
**Ways of COVID-19-related work**					
Volunteer (*n* = 726)	96 (13.2)/80 (11.0)	7.330*/34.911**			
Designated (*n* = 212)	44 (20.8)/58 (27.4)				

*p < 0.05; **p ≤ 0.001.

### Correlation analysis

**FIGURE 1:**
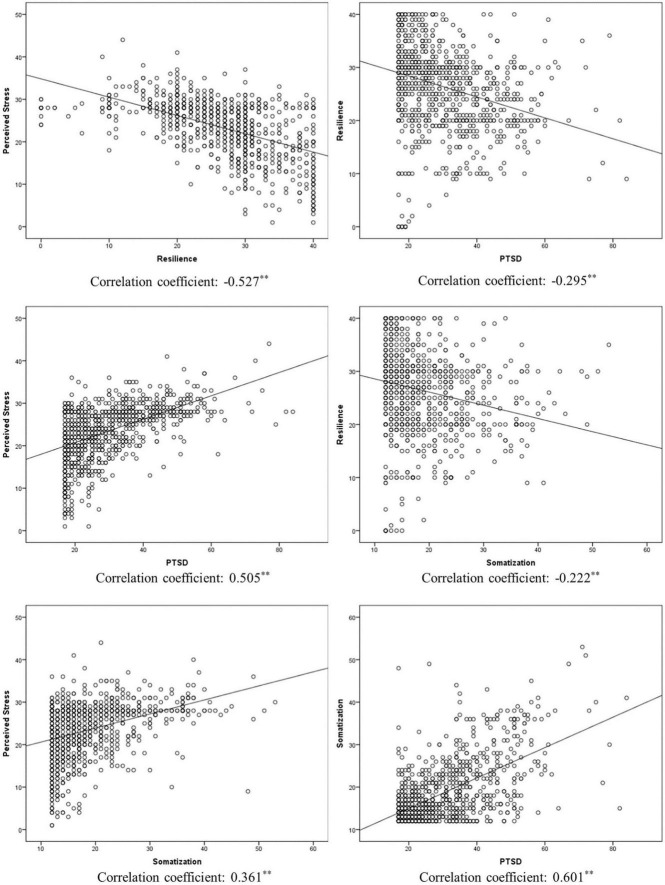
Scatter plots for study variables (^**^p < 0.001).

All study variables were normally distributed, and the mean and standard deviation were used to express the data. As shown in Table 4, resilience was negatively correlated with all other variables. Perceived stress was positively correlated with PTSD (*r* = 0.505, *p* < 0.001) and somatization (*r* = 0.361, *p* < 0.001). Scatter plots of study variables with fit lines are demonstrated in Figure 1.

**TABLE 4:** Correlation between the study variables (*n* = 938).

	M ± *SD*	2	3	4
1. Resilience	26.49 ± 8.13	–0.527**	–0.295**	–0.222**
2. Perceived stress	23.41 ± 6.64	–	0.505**	0.361**
3. PTSD	29.66 ± 12.19	–	–	0.601**
4. Somatization	18.45 ± 7.26	–	–	–

**p < 0.001.

### Mediation model

The mediatory role of perceived stress in the relationship between resilience and PTSD/somtization was examined. The mediation effects are shown in Table 5. Regarding the dependent variables of PTSD, the results revealed a significant indirect effect of resilience on PTSD (*b* = –0.382, bootstrapped 95% CI = –0.454, –0.319). However, resilience did not have significant direct effects on PTSD in the presence of the mediator (*b* = –0.060, *p* = 0.226). These results suggest that perceived stress mainly mediates the relationship between resilience and PTSD.

**TABLE 5:** Direct, indirect, and total effects of the mediator.

Predictor variable (X)	Mediator variable (M)	Result variable (Y)	Effect of X on M (a)	Effects of M on Y (b)	Direct effect (c´)	Indirect effect (a*b)	Bootstrapped 95% CI	Total effect (c)
Resilience	Perceived stress	PTSD	–0.431**	0.887**	–0.060	–0.382	–0.454, –0.319	–0.442**
		Somatization	–0.431**	0.370**	–0.039	–0.159	-0.199, –0.123	–0.198**

5,000 resamples. PTSD, Post-traumatic stress disorder. **p < 0.01.

**FIGURE 2:**
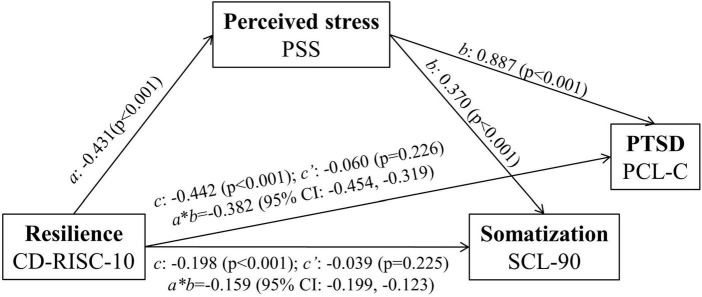
Mediation analysis exploring the relationship among resilience, PTSD, and somatization. c’, direct effect; c, total effect; (a*b), indirect effect. After monitoring the effects of covariates (age, sex, educational qualification, income, marital status, staff, title, years of work, worksite, work content, provision of support to Hubei in 2019, work schedule/week, ways of COVID-19-related work, training, competence in position, mental support, frequency of contact with family and friends, and psychological support from family and friends), the analysis of mediation models showed that the total effect of resilience on PTSD and somatization remained significant (PTSD: c = -0.433, *p* < 0.001; somatization: c = -0.170, *p* < 0.001). The direct effect (c’) of resilience associated with the mediator (PTSD: c’ = -0.086; somatization: c’ = -0.030) was altered but remained insignificant after controlling the variance. These results suggest that perceived stress mainly mediates the association between PTSD and somatization through resilience.

Regarding the dependent variables of somatization, the results revealed a significant indirect effect of resilience on somatization (*b* = –0.159, bootstrapped 95% CI = –0.199, –0.123). However, resilience did not have significant direct effects on somatization in the presence of the mediator (*b* = –0.039, *p* = 0.225). These results suggest that perceived stress mainly mediates the relationship between resilience and somatization. The results of the final model are shown in Figure 2.

## Discussion

Fighting against COVID-19 is extremely stressful and affects the mental and physical wellbeing of frontline healthcare professionals. Therefore, it is important to diagnose PTSD symptoms, somatization, and perceived stress in frontline healthcare professionals to enable prompt and effective intervention. In this study, we evaluated the prevalence of resilience, perceived stress, PTSD, and somatization among frontline healthcare professionals involved in the treatment/management of COVID-19 and examined the mediatory role of perceived stress in PTSD and somatization. The prevalence rate of PTSD was 14.9%, whereas that of moderate- or higher-degree somatization was 14.72% among frontline healthcare professionals. In addition, perceived stress was found to mediate the relationship between resilience and PTSD/somatization.

PTSD and somatization are severe psychological problems that negatively affect the quality of life of frontline healthcare professionals. In this study, approximately 14.9% of frontline healthcare professionals had PTSD, and the top five somatic symptoms in 54.05% of frontline healthcare professionals included trouble falling asleep, fatigue, insomnia, inability to wake up early, and muscle soreness. These findings are consistent with those of previous studies, which reported that 13.7% of healthcare professionals (33) and 16.83% of nurses (34) involved in the management of COVID-19 had PTSD. However, this proportion is higher than that of healthcare professionals working in hospitals with established fever clinics or wards for patients with COVID-19 [9.1% (35) and 9.8% (36)]. In this study, we only included frontline healthcare professionals who cared for patients with COVID-19 and individuals at risk for COVID-19. The nature of working in medical wards and the uncertainty of contact with the risk population can increase stress and promote PTSD. This study showed that healthcare professionals who volunteered to work on the frontline, were engaged in non-clinical treatment/caring, practiced self-assessment to be competent in their medical position, had frequent contact with family and friends, and received psychological support from family and friends were less likely to have PTSD symptoms. Healthcare professionals who volunteer and self-assessment to be competent are better prepared to fight against challenges compared with those designated to work on the frontline. Healthcare professionals engaged in clinical treatment/caring are in close contact with patients with COVID-19, which poses great psychological threat. Healthcare professionals who have frequent contact with and receive psychological support from family and friends are well-equipped to cope with stress.

As reported in a study on Chinese healthcare workers, the prevalence of somatization among healthcare workers during the COVID-19 pandemic was 9.59%, which is slightly lower than that observed in this study, possibly owing to the recruitment of frontline healthcare professionals in this study (19). The nature of frontline healthcare work increases the risk of infection because of continued exposure to COVID-19 and excessive focus on disease symptoms (37). In this study, sleep-related problems were the most common symptoms among frontline healthcare professionals, which is consistent with the results of a previous study, indicating that the prevalence of poor sleep quality increased among healthcare professionals during the COVID-19 pandemic (38). Similar results were obtained in univariate analysis for somatization. Healthcare professionals who did not volunteer to work on the frontline, did not receive training on the treatment and caring of patients with COVID-19, were not competent in their position, did not receive mental support, less frequently contacted their family and friends, and did not receive psychological support from family and friends were more like to have a moderate or higher degree of somatization.

Furthermore, perceived stress was found to mediate the association between resilience and PTSD/somatization. Resilience reflects an individual’s ability to withstand hardship and adapt to changes caused by stressful events in a flexible way (39). Individuals with higher resilience are equipped with better coping skills, which is further associated with a low level of perceived stress. Perceived stress reflects the feelings or thoughts that individuals have about how much stress they are experiencing and the degree to which the situation is considered stressful at a given time point or within a specific period (40). Consequently, it may affect the symptoms of PTSD and somatization. A similar phenomenon was observed in a previous study, which reported that perceived stress mediates the relationship between resilience and somatization in teachers, students, and healthcare workers (21). In addition, perceived stress can mediate the association between social adaption and quality of life in individuals at ultra-high risk for psychosis (41). Social adaption reflects resilience, whereas the quality of life reflects mental wellbeing. Therefore, resilience can help individuals to improve their quality of life by reducing perceived stress (41). In this study, a high proportion of healthcare professionals had low resilience, which impacted their mental health. Therefore, novel strategies for building resilience should be developed to help frontline healthcare professionals fight against negative mental outcomes.

To the best of our knowledge, a few studies have examined the mediating effects of perceived stress on the relationship between resilience and PTSD and between resilience and somatization among frontline healthcare professionals in China. This study offers insights into the prevention and alleviation of PTSD and somatization. Low resilience and high levels of perceived stress may be the markers of vulnerability to PTSD and somatization. Therefore, efforts should be made to increase resilience and reduce perceived stress to decrease the incidence of PTSD and somatization. Stress perception can be promoted by some techniques, such as reduction in mind–body stress *via* breath work, yoga, and meditation (42). In addition, increased resilience is associated with decreased vulnerability to stress, thus preventing PTSD and somatization. Therefore, future studies should be conducted to develop strategies for building resilience by improving stress perception for the prevention and treatment of PTSD and somatization among frontline healthcare workers involved in the management of COVID-19.

This study has several potential limitations. The main limitation is the cross-sectional design, which prevents from inferring causality in the relationship between resilience and PTSD and between resilience and somatization. In addition, this study was confined to a specific region (Shaanxi), therefore, the results cannot be generalized.

## Conclusion

Some frontline healthcare professionals may have low resilience and hence a predisposition to PTSD. In addition, perceived stress mediates the relationship between resilience and PTSD/somatization. Therefore, efforts should be made to increase resilience and alleviate stress among healthcare professionals to decrease the incidence of PTSD and somatization.

## Data availability statement

The raw data supporting the conclusions of this article will be made available upon reasonable request.

## Ethics statement

The studies involving human participants were reviewed and approved by the Shaanxi Provincial People’s Hospital Ethics Committee. The patients/participants provided their written informed consent to participate in this study.

## Author contributions

ML: conception of the study, data analysis, and drafting of the manuscript. XY: conception of the study and critical review and revision of the manuscript. DW, YW, LY, and YM: data collection and review and revision of the manuscript. XL: data collection and review of the manuscript. YZ: study design and approval of the submitted version of the manuscript. All authors contributed to this study and approved the final version of the manuscript.

## Footnotes

1
www.wjx.cn
